# The GWAS Analysis of Body Size and Population Verification of Related SNPs in Hu Sheep

**DOI:** 10.3389/fgene.2021.642552

**Published:** 2021-05-20

**Authors:** Junfang Jiang, Yuhao Cao, Huili Shan, Jianliang Wu, Xuemei Song, Yongqing Jiang

**Affiliations:** ^1^Institute of Animal Husbandry and Veterinary, Zhejiang Academy of Agricultural Sciences, Hangzhou, China; ^2^Zhejiang Key Laboratory of Pathophysiology, Department of Biochemistry and Molecular Biology, Medical School of Ningbo University, Ningbo, China

**Keywords:** Hu sheep, body size traits, genome wide association studies, SNPs, transcription activity, population verification

## Abstract

Body size is an important indicator of growth and health in sheep. In the present study, we performed Genome-Wide Association Studies (GWAS) to detect significant single-nucleotide polymorphisms (SNPs) associated with Hu sheep’s body size. After genotyping parental (G1) and offspring (G2) generation of the nucleus herd for meat production of Hu sheep and conducting GWAS on the body height, chest circumference, body length, tail length, and tail width of the two groups, 5 SNPs associated with body height and 4 SNPs correlated with chest circumference were identified at the chromosomal significance level. No SNPs were significantly correlated to body length, tail length, and width. Four out of the 9 SNPs were found to be located within the 4 genes. *KITLG* and *CADM2* are considered as candidate functional genes related to body height; *MCTP1* and *COL4A6* are candidate functional genes related to chest circumference. The 9 SNPs found in GWAS were verified using the G3 generation of the nucleus herd for meat production. Nine products were amplified around the 9 sites, and 29 SNPs were found; 3 mutation sites, G > C mutation at 134 bp downstream of s554331, T > G mutation at 19 bp upstream of s26859.1, and A > G mutation at 81 bp downstream of s26859.1, were significantly correlated to the body height. Dual-luciferase reporter gene experiments showed that the 3 SNPs could significantly impact dual-luciferase and gene transcription activity.

## Introduction

In sheep, body size has been widely recognized as an important indicator of growth and health (Kemper et al., 2012), which impacts animal feeding and management as well as adaptation to the environment. Mature body size has been extensively studied in humans, cattle, and other domestic animals but not in sheep (Posbergh and Huson, 2021). In sheep, the mature body size is more polygenic than in other domesticated animals, which suggests that the development of genomic trait selection might be the optimal option for evaluating body size in sheep (Posbergh and Huson, 2021).

GWAS (Genome-wide association study) is a method that uses millions of single nucleotide polymorphism (SNP) in genomes as molecular genetic markers to conduct control analysis or correlation analysis at the whole genome level so as to investigate the genetic mutation of complex traits. This technique has been applied to screen the SNPs of agricultural animals’ major traits.

Eight common gene candidates, i.e., *GRID1*, *ALOX12*, *SLC16A13*, *SLC16A11*, *TP53*, *STX8*, *NTN1*, and *ZNF521*, were identified from GWAS for body size traits in crossbreeding sheep between Frizarta sheep and East Friesian sheep (Kominakis et al., 2017). In Hulun Buir sheep, 13 candidate genes, including *SMURF2*, *FBF1*, *DTNBP1*, *SETD7*, and *RBM11*, have been associated with fat metabolism, and *SMARCA5* and *GAB1* were associated with body size (Zhang et al., 2019). In addition, *MARCA5* and *GAB1* have been found to be related to sheep’s body size. Height has been associated with 12 SNPs across six chromosomes. Ear length was associated with a single locus on chromosome 3.

Hu sheep, which are mainly housed all year round, are a special type of sheep that is only found in China. Hu sheep are characterized by early sexual maturity, high fecundity, and rapid growth. It is famous for its beautiful lamb skin. Hu sheep also have good meat quality, strong resistance to stress, and resistance to rough feeding (Yue, 1996). Until now, no GWAS study on the body size traits of Hu sheep has been reported.

In this study, GWAS were applied to screen and select candidate SNPs for traits and body size of meat-type Hu sheep. Moreover, the candidate SNPs associated with Hu sheep’s body size were verified among meat-type Hu sheep’ offspring of G3 generation.

## Materials and Methods

### Animals

The GWAS study included 240 Hu sheep from G1 and G2 generation of meat-type Hu sheep nucleus herd in Huzhou Taihu Lake Culture Cooperative by semi-open nucleus breeding. The SNP herd verification included 202 Hu sheep from the G3 generation of Hu sheep nucleus herd in Hangzhou Pangda Agricultural Development Co., Ltd. The breeding and management of the Hu sheep included in the study were conducted according to the standard methods of breeding and management of Hu sheep.

### Determination of Body Size Traits and Genomic DNA Extraction

Selected body size traits included body height, chest circumference, body length, tail length, and tail width. The body height and body length were measured using a measuring stick (Zhengzhou Zhimuren Machinery Equipment Co., Ltd.) with an accuracy of 1 mm; chest circumference, tail length, and tail width were measured using a tape measure with an accuracy of 1 mm (Zhengzhou Zhimuren Machinery Equipment Co., Ltd.). Sheep were on horizontal ground, quiet and relaxed. The measurement methods were: (1) body length: the straight-line length from the front edge of the shoulder and foot bones to the back edge of the ischial tuberosity; (2) body height: the vertical length from the highest point of the bun to the ground; (3) chest circumference: the length of the circumference around the back edge of scapula; (4) tail length: the length from root to the top of the tail; (5) tail width: the widest range length of the tail. All measurements were performed by the same worker to minimize measurement errors caused by artificial reasons. Each sheep was measured at least 2 times, and the average was taken as the final measurement result. In addition, a total of 10 ml of blood was collected from each sheep’s jugular vein and then placed into EDTA anticoagulant tubes. DNA was extracted with phenol/chloroform extraction method and kept at −20°C.

### Genotyping and Quality Control

Ovine SNP50 BeadChip was applied to genotype individual SNPs. The chip was co-developed by Illumina and experts from International Sheep Genomics Consortium. Plink 1.09 software was applied to conduct quality control on genotypes, phenotypic data and samples, analyze SNPs, and estimate genotypes and phenotypic value.

### Population Structure Analysis and Genome-Wide Association Study

Population structure analysis was performed using admixture v1.3. A heat map of the values in the kinship matrix was created for the kinship plot. After quality control was performed on genotype data, GWAS on SNP was performed using the mixed linear model (MLM) of TASSEL5.0 software to identify SNPs related to the body size traits of the nucleus herd for meat production of Hu sheep. MLM model was adjusted according to 3 confounding factors, i.e., sex, herd structure, and genetic relationship. The following concrete model was used:

Y=X⁢β+S⁢α+Q⁢v+Z⁢u+e

where Y is the phenotypic value of Hu sheep’s body size traits; β stands for fixed effects apart from SNP and herd structure; α stands for SNP effect; v represents herd structure effect; u stands for polygenic background effect; e represents residual effect, and X, S, Q, Z represent the incidence matrix of β, α, v, u, respectively.

When performing correlation analysis on the body size traits of Hu sheep, if errors were found in multiple hypothesis tests, a *p-*value was analyzed and adjusted. MLM was used to calculate F and *p* values, after which the results were verified. The following formula was applied:

P⁢s=α/N,

where α stands for the level of significance and *N* for the number of independent SNPs used in the analysis. If the *p*-value at the SNP site was less than α, this SNP site was considered as significantly correlated to body size traits.

After SNPs sites were obtained with the performance of GWAS, base sequence 500 bp upstream and downstream of the significantly correlated SNP sites were downloaded. Next, BLAST research for the sequence was then performed with NCBI and Ovis aries_v4.0 (UCSC) to confirm the information of the location of SNP and adjacent genes.

### Group Verifying of Significantly Correlated SNPs

Two hundred two ewes (from Hangzhou Pangda Agricultural Development Co., Ltd.) were included as subjects. PCR Amplification, product sequencing, and gene sequence analysis were used to perform SNPs detection. Base sequence 500 bp upstream and downstream of the SNP sites were significantly correlated to Hu sheep’s body height, and chest circumference was downloaded. Primers are shown in Supplementary Table 1. A total of 25 μL PCR reaction system was used for PCR amplification; the reaction procedure included: initial denaturation for 2 min at 94°C; denaturation for 30 s at 95°C, annealing for 30 s at 55°C, an extension for 30 s at 72°C, 35 circulations; extension for 10 min at 72°C, preservation at 4°C after the completion of the reaction. Direct sequencing was performed on the upstream and downstream primers for PCR products for each SNP site of each sample. Mutation Surveyor 5.02 was used to analyze the forward and reverse sequencing diagram of each ewe so as to confirm the mutation sites and mutation methods of the sequencing results of the amplified products at different sites in each sample. PopGen32 was used to calculate the gene frequency and genotype frequency of the SNPs. Hardy-Weinberg equilibrium test was performed to calculate Polymorphism information content (PIC).

The relationship between the different genotypes or haplotypes and body size traits of meat-type Hu sheep was evaluated by fitting a general linear model using the restricted maximum likelihood method in the Statistical Package for the Social Sciences (SPSS; version 20.0; SPSS Inc., Chicago, IL, United States). The general model used for Hu sheep body size traits was:

Y⁢i⁢j=μ⁢i+M⁢j+e⁢i⁢j

where Yij is the meat type Hu sheep body height or Chest circumference; μi is the least square mean; Mj is the fixed effect of the jth genotype or haplotypes, and eij is the random residual effect of each Hu sheep body height or chest circumference value.

### Linkage Disequilibrium Analysis

HaploView version 4.2 was used to perform Linkage disequilibrium (LD) block and Haplotype analyses (Whitehead Institute for Biomedical Research, Cambridge, MA, United States). The *D*′-value of the lower 95% confidence interval in the analysis was used to define the haplotype block (Brym et al., 2005).

### Effects of Candidate Functional SNPs or Haplotypes on Gene Transcriptional Activity

Candidate functional SNP loci significantly associated with body height in the wild-type and homozygous mutant sheep were selected. The different haplotypes amplification product was cloned into the pGL4.10 vector (Promega, United States), expressing a dual-luciferase gene (General Biosystems Corporation, Anhui, China). The vector was then transfected into sheep kidney cells. After 24 h, the luciferase activity was measured on a microplate reader using the Dual-Luciferase^®^ reporter assay system (Promega, United States).

The relationship between the different genotypes or haplotypes and luciferase activities was evaluated by fitting a general linear model using the restricted maximum likelihood method in the Statistical Package for the Social Sciences (SPSS; version 20.0; SPSS Inc., Chicago, IL, United States). The general model used for luciferase activity value was:

Y⁢i⁢j=μ⁢i+M⁢j+e⁢i⁢j

where Yij is the luciferase activities; μi is the least square mean; Mj is the fixed effect of the jth genotype or haplotypes, and eij is the random residual effect of luciferase activities.

## Results

### Descriptive Statistics and Quality Control

The descriptive statistical information of the phenotypic values related to the individual body size traits of 240 G1 and G2 generation Hu sheep has is shown in Supplementary Table 2. The descriptive statistical information of the phenotypic values related to the individual body size traits of 202 G3 generation Hu sheep is shown in Supplementary Table 3.

In the previous work, we focused on Genome-Wide Association Study of body weights in Hu Sheep. Here, we examined the relationship between the same population and body sizes traits. The result of data quality control can be found in the study by Cao et al. (2020).

### Population Structures and Association Analyses

**FIGURE 1:**
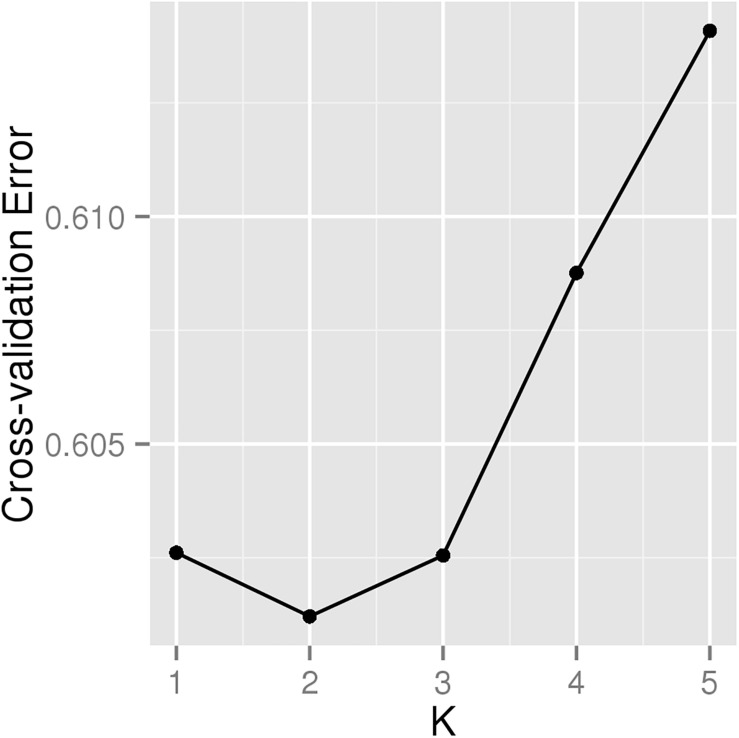
Cross-validation plot for determining the best K.

**FIGURE 2:**
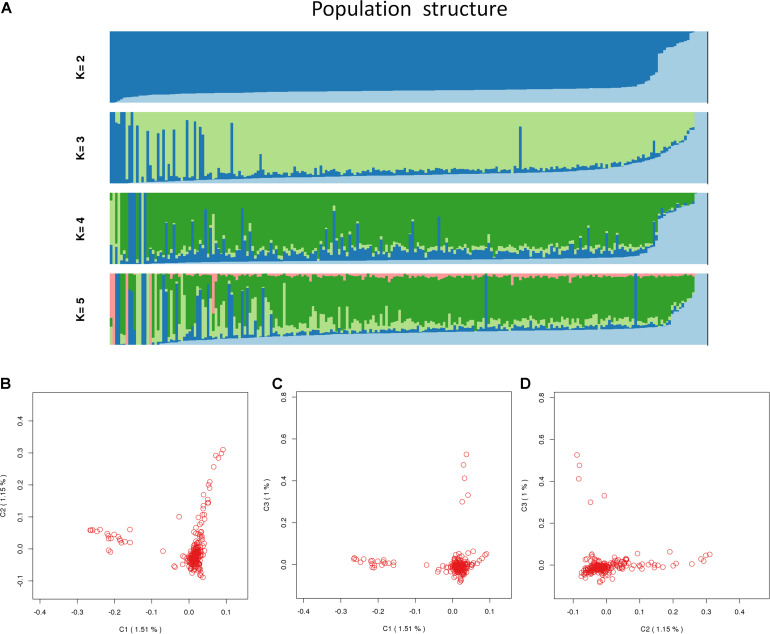
Population_structure_by_Admixture. **(A)** Population structure with K from 2 to 5; **(B)** principal components (PC) plot drawn from the second principal component (C2) against the first principal component (C1); **(C)** principal components (PC) plot drawn from the second principal component (C3) against the first principal component (C1); **(D)** principal components (PC) plot drawn from the second principal component (C3) against the first principal component (C2).

A total of 226 Hu sheep were randomly selected from the group of Huzhou Taihu Lake Culture Cooperative. According to the population structure, the result was given by admixture v1.3 with K from 1 to 5, where the optimal K was 2 (Figure 1). Kinship estimation and Principle component analysis (PCA) of all individuals indicated the effectiveness of sampling (Figures 2A–D).

**FIGURE 3:**
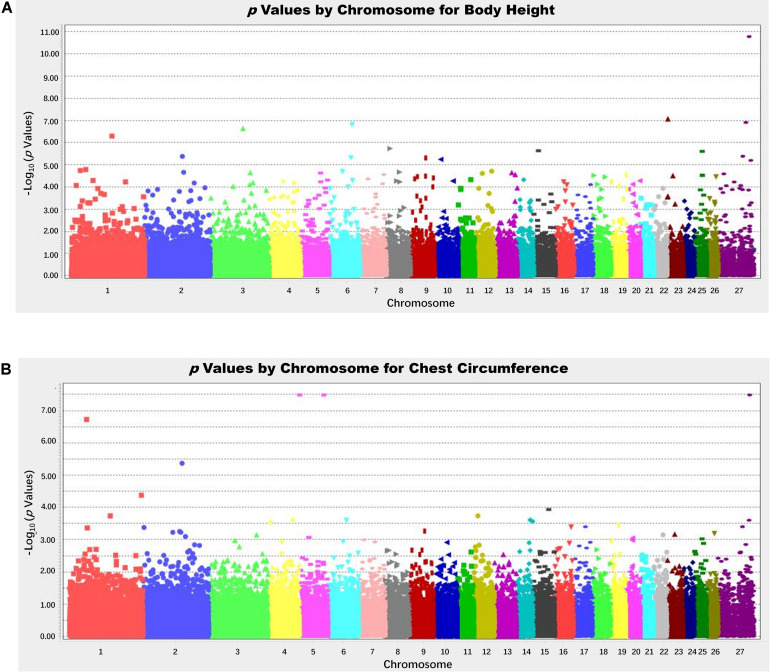
Manhattan plot of analysis results; -log_10_ (*p*-values) in the studied population of Hu sheep. **(A)** Manhattan plot of the results of the body height analysis. **(B)** Manhattan plot of the results of the chest circumference.

Based on the number of independently effective SNPs, the *p*-value corresponding to the 1% significance level was 2.83 × 10^–7^, and that corresponding to the 5% significance level was 1.41 × 10^–6^. SNPs with a *p*-value lower than this threshold value were considered to be significantly correlated to phenotype. GWAS’ results showed that 5 SNPs were significantly correlated to body height (Figure 3) in terms of genomic level; OAR23_3237800.1 (*p* = 5.53 × 10^–8^) of chromosome 23; OAR6_95218086.1 (*p* = 1.52 × 10^–8^) of chromosome 6; OARX_120998827.1 (*p* = 1.22 × 10^–7^) of chromosome 27 and OAR3_132833292.1 (*p* = 2.30 × 10^–7^) of chromosome 3; OAR1_164254640.1 (*p* = 5.08 × 10^–7^) (Figure 3) of chromosome 1. Four SNPs, s55433.1 (*p* = 3.26 × 10^–8^) and OAR5_99879334.1 (*p* = 3.26 × 10^–8^) of chromosome 5, OARX_79209204.1 (*p* = 3.26 × 10^–8^) of chromosome 27, and s26859.1 (*p* = 1.89 × 10^–7^) of chromosome 1 (Table 1), were significantly correlated to chest circumference (Figure 3) in terms of genomic level; no SNP was significantly correlated to body length, tail width or tail length.

**TABLE 1:** Analysis of related SNPs associated with body size traits of Hu sheep.

**Trait**	**Related SNPs**	**Chr**	**Position (bp)**	***p*-value**	**add_effect *p*-value**	**Nearest gene distance^#^ (bp)**
Body height	OAR23_3237800.1	23	2959015	5.53E-08	9.21E-08	*ZNF516* -368856
	OAR6_95218086.1	6	86778759	1.52E-08	3.06E-08	*NPFFR2* + 114271
	OARX_120998827.1	27	105184061	1.22E-07	1.75E-08	*PRR32* + 10161
	OAR3_132833292.1	3	124516955	2.30E-07	NaN	*KITLG* within
	OAR1_164254640.1	1	152601830	5.08E-07	NaN	*CADM2* within
Chest circumference	s55433.1	5	413256	3.26E-08	NaN	*LOC101119639* + 63923
	OAR5_99879334.1	5	91556623	3.26E-08	NaN	*MCTP1* within
	OARX_79209204.1	27	119635739	3.26E-08	NaN	*COL4A6* within
	s26859.1	1	63255194	1.89E-07	NaN	*SELENOF* + 22053

*p-values calculated from the mixed linear model analysis.^#^Positive value denotes the gene location downstream of SNP; negative value denotes the gene location upstream of SNP.*

At the genomic level, annotation information of the 9 SNPs sites that were significantly correlated to body height and chest circumference are shown in Table 1. Four of the SNPs were within genes. OAR3_132833292.1 was within gene *KITLG*; OAR1_164254640.1 was within gene *CADM2*; OAR5_99879334.1 was within gene *MCTP1*; OARX_79209204.1 was within gene *COL4A6*. There were 5 other SNPs at intergenic regions: OAR23_3237800.1 located 368,856 bp downstream of *ZNF516*, OAR6_95218086.1 located 114,271 bp upstream of *NPFFR2*, OARX_120998827.1 located 10,161 bp upstream of *PRR32*, s55433.1 located 63,923 bp upstream of LOC101119639, and s26859.1 located 22053bp upstream of *SELENOF* (Table 1).

### Group Verifying of SNPs Significantly Correlated to Hu Sheep’s Body Height

**FIGURE 4:**
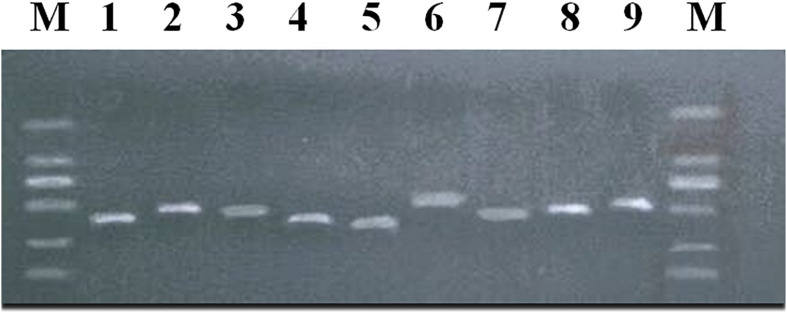
PCR amplification products of body size-related SNPs in meat-type Hu sheep. M:DL2000 plus. (1) *LOC101119639* + 63923; (2) *MCTP1*; (3) *COL4A6*; (4) *ZNF516* -368856; (5) *SELENOF* + 22053; (6) *KITLG*; (7) *NPFFR2* + 114271; (8) *CADM2*; (9) *PRR32* + 10161.

The above 9 SNPs sites found by GWAS were verified by using the G3 generation 202 ewes of the nucleus herd for meat production. Nine products were amplified around nine sites (Figure 4), and 29 SNPs were found. Two mutation sites were detected in the amplified products at s55433.1, two mutation sites in the amplified products at OAR5_99879334.1, five in the amplified products at OARX_79209204.1, three in the amplified products at OAR23_3237800.1, three in the amplified products at s26859.1, six in the amplified products at OAR3_132833292.1, two in the amplified products at OAR6_95218086.1, two in the amplified products at OARX_120998827.1, and four mutation sites were detected in the amplified products at OAR1_164254640.1 (Supplementary Table 4).

Population genetic analysis was performed on 29 sites. All loci were subjected to genotyping, population genetic analysis, and the association analysis between SNPs and body size traits. The value of PIC at 24 sites was < 0.25, which was indicative of low polymorphism. The value of PIC at five sites was between 0.25 and 0.5, which was indicative of intermediate polymorphism (Supplementary Tables 5, 6).

Verification results of SNPs showed that three mutation sites were significantly correlated to Hu sheep’s body height (Table 2): G > C mutation at 134 bp downstream of s554331, T > G mutation at 19 bp upstream of s26859.1, A > G mutation at 81 bp downstream of s26859.1.

**TABLE 2:** Association analysis between SNPs and body height of Hu sheep.

**SNP loci**	**Nearest gene distance^#^ (bp)**	**Chr**	**Position (bp)**	**Genotype**	**Numbers**	**Body height (kg)**
G > C mutation at 113 bp downstream of s554331	*LOC101119639* + 63789	5	413,369	CC	49	75.22 ± 0.43^b^
				GG	153	76.49 ± 0.24^a^
				*p*-value		0.011
T > G mutation at 19 bp upstream of s26859.1	*SELENOF* + 22072	1	63,255,175	GG	3	72.67 ± 1.74^b^
				TG	51	75.53 ± 0.42^b^
				TT	148	76.48 ± 0.25^a^
				*p*-value		0.021
A > G mutation at 81 bp downstream of s26859.1	*SELENOF* + 21972	1	63,255,275	AA	154	76.46 ± 0.24^a^
				AG	46	75.54 ± 0.44^a^
				GG	2	70.00 ± 2.15^b^
				*p*-value		0.003

*Values with different superscripts for the same column have significant differences.*

### Linkage Disequilibrium and Haplotype Block Analyses

**FIGURE 5:**
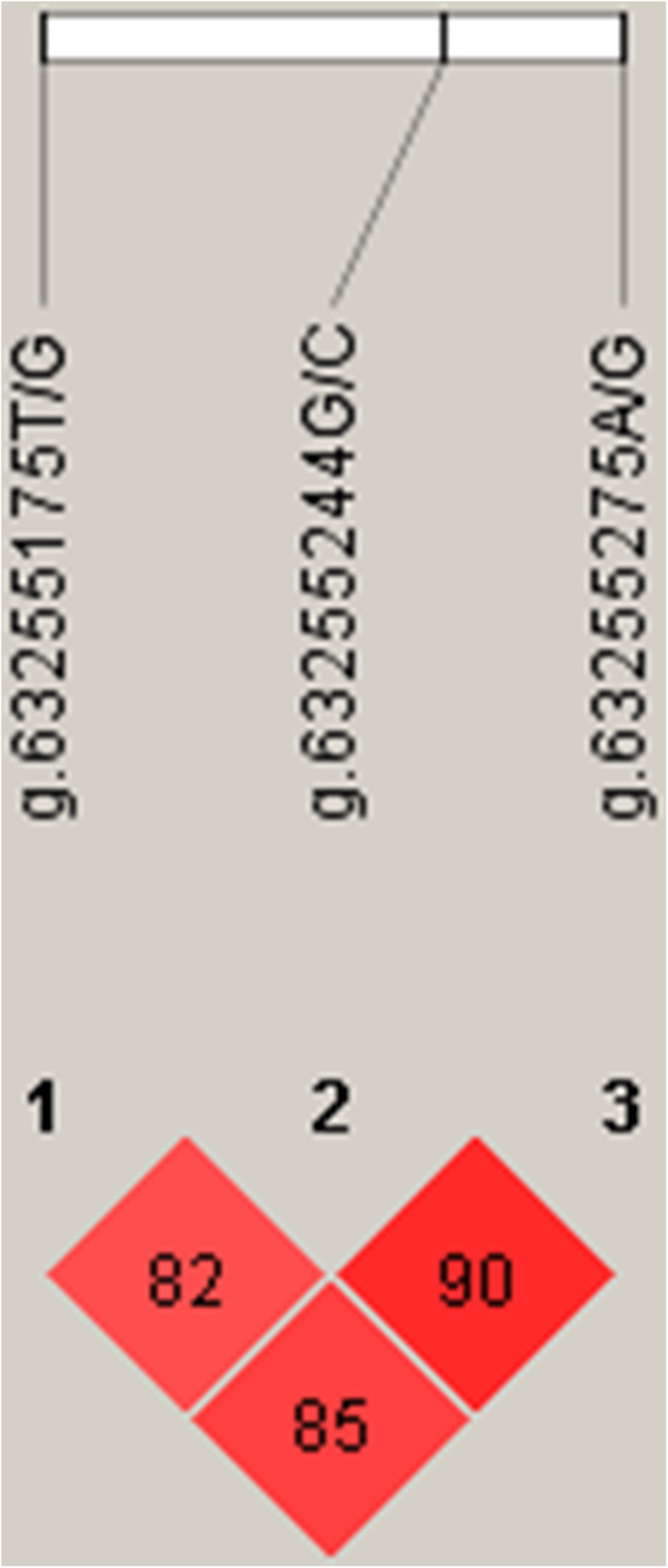
Linkage disequilibrium (LD) analyses of SNPs near s26859.1.

Linkage disequilibrium (Linkage Disequilibrium, LD) refers to the non-random co-occurrence of alleles of chromosomes or haplotypes, i.e., there are statistical associations between alleles at different sites, which are different from independent alleles. Usually, D′ and r^2^ are used to measure LD. D′ > 0.33 and *r*^2^ > 0.1 represent a meaningful linkage disequilibrium; *D*′ > 0.8 and *r*^2^ > 0.33 a strong linkage disequilibrium (Long et al., 2004). According to the LD analysis results, the three SNPs showed strong linkage disequilibrium (Figure 5).

Three SNPs, g.63255175T/G, g.63255244G/C, and g.63255275A/G, were chosen for haplotype analysis based on linkage disequilibrium evaluation (*D*′ > 0.8, *r*^2^ < 0.05). Two tag SNPs (g.63255175T/G, g.63255275A/G) represented the genetic variation in the haplotype block. The effects of different haplotypes indicated a significant effect of haplotypes on the body height of Hu sheep (Table 3).

**TABLE 3:** Association analysis between haplotypes and body height of Hu sheep.

**Loci**	**Haplotypes**	**Numbers**	**Body height (cm)**
	GGCCAA	1	78.00^ab^
	GGCCGG	2	70.00 ± 5.66^c^
	TGCCAA	8	74.00 ± 3.21^bc^
T > G mutation at 19 bp upstream of s26859.1	TGCCAG	32	75.50 ± 2.44^b^
	TGGCAA	2	79.00 ± 0.00^ab^
G > C mutation at 50 bp downstream of s26859.1	TGGCAG	7	75.57 ± 2.70^b^
	TGGGA A	1	82.00^a^
A > G mutation at 81 bp downstream of s26859.1	TGGGAG	1	75.00^bc^
	TTCCAA	56	76.27 ± 3.22^ ab^
	TTCCAG	6	75.83 ± 3.37^ ab^
	TTGCAA	69	76.71 ± 2.96^ ab^
	TTGGAA	17	76.47 ± 2.79^ ab^

*Note: Values with different superscript for the same column have significant differences.*

### Effects of Candidate Functional SNPs on Gene Transcriptional Activity

The results of dual-luciferase reporter gene experiments (Table 4) showed that SNPs (G > C mutation at 134 bp downstream of s554331) significantly impacted the activity of dual-luciferase and decreased the activity of dual-luciferase after mutation (*p* < 0.05). Moreover, SNPs (T > G mutation at 19 bp upstream of s26859.1; A > G mutation at 81 bp downstream of s26859.1) significantly decreased the activity of dual-luciferase after mutation (all *p* < 0.05). Haplotypes (T > G mutation at 19 bp upstream of s26859.1, A > G mutation at 81 bp downstream of s26859.1) significantly affected the activity of the reporter gene (*p* < 0.05). These results showed that the above SNPs and haplotypes could significantly impact the activity of gene transcription.

**TABLE 4:** Effects of genotypes or haplotypes of candidate functional SNPs on dual-luciferase activities.

**SNPs locus**	**SNPs genotypes or haplotypes**	**M1/M2**	***p*-value**
G > C mutation at 134 bp downstream of s554331	GG	4.26 ± 0.44^a^	0.009
	CC	2.14 ± 0.07^b^	
T > G mutation at 19 bp upstream of s26859.1	TT	1.91 ± 0.53^b^	0.000
	GG	4.53 ± 2.21^a^	
A > G mutation at 81 bp downstream of s26859.1	AA	4.45 ± 2.30^a^	0.000
	GG	1.99 ± 0.61^b^	
T > G mutation at 19 bp upstream of s26859.1	TTAA	2.37 ± 0.08^b^	0.000
	GGAA	6.53 ± 0.49^a^	
A > G mutation at 81 bp downstream of s26859.1	TTGG	1.45 ± 0.24^c^	
	GGGG	2.53 ± 0.10^b^	

*Note: Values with different superscript for the same column have significant differences.*

## Discussion

Existing GWAS studies on sheep have mainly focused on reproductive traits (Demars et al., 2013; Gholizadeh et al., 2014; Martinez-Royo et al., 2017; Abdoli et al., 2019), body weight, and meat production traits (Zhang et al., 2013; Almamun et al., 2015; Matika et al., 2016), while few investigated body size traits. Thus far, no SNPs significantly correlated to body size traits at the genomic level were identified at *p* < 0.05. Eleven chromosome-wide significant SNPs, five for the “width Dimension” factor, four for the “height Dimension” factor, and two for the “length Dimension” factor were confirmed at *p* < 0.10 (Kominakis et al., 2017). One SNP (OAR17_14085599) was found to be significantly correlated to chest circumference. No SNPs were found to be correlated to body length and height (Zhang et al., 2019). In the present study, we found that 5 SNPs were significantly correlated to body height, and 4 SNPs were significantly correlated to chest circumference. Compared to the previous two studies, these SNPs were significantly different.

Our results identified nine significant SNPs at the genomic level. After performing genome annotation on the 9 SNPs, some candidate functional genes correlated to Hu sheep’s body height and chest circumference were found: *KITLG* and *CADM2* are candidate functional genes correlated to body height, while *MCTP1* and *COL4A6* are candidate functional genes correlated to chest circumference.

OAR1_164254640.1 is within *CADM2* (Gene ID: 101120371). Cell adhesion molecules (*CADM*) consist of a protein family that maintains cell polarity. Most *CADM* belong to the immunoglobulin superfamily. Previous studies have shown that *CADM* can be used as a tumor inhibitor (He et al., 2013). *CADM2* belongs to the *CADM* family. *CADM2* activates methylation and/or heterogeneity loss by promoting DNA to contain human kidney clear cell carcinoma. The loss of *CADM2* leads to tumor progression (He et al., 2013). Previous genome-wide association meta-analysis confirmed several susceptibility sites to be correlated to BMI, including *CADM2* (Speliotes et al., 2010; Locke et al., 2015). Moreover, obesity and glucose level can be reduced, and insulin sensitivity, sports function, energy expenditure rate, and core temperature can be increased in cadm2-knockout mice, emphasizing its relevance in systematic energy balance (Yan et al., 2018). Moreover, *CADM2* is related to a series of behavioral and metabolic features, including physical activity, adventure, educational level, and obesity (Morris et al., 2019). It has been proved that *CADM2* gene mutation has a critical role in BMI through the central nervous system (Speakman et al., 2018).

OAR5_99879334.1 is within gene *MCTP1*, a neuronal vesicle/endosome protein. In terms of structure, *MCTP* protein contains 3 C2 domains and 2 transmembrane domains near the C-end (Shin et al., 2005). The mutation or expression of *MCTP1* variants is related to neuro psychosis. Genome-wide analysis shows that *MCTP1* single nucleotide polymorphism (SNP), rs17418283, is related to bipolar affective disorder (Scott et al., 2009). *In vivo* and *in vitro* imaging studies all identified the location of *MCTP1* on the endocrine recovery approach. Moreover, functional tests have shown that *MCTP1* participates in various cell functions, including endocrine, cell migration, and anti-excitement virulence of neuronal cells (Qiu et al., 2015).

OAR3_132833292.1 is within the *KITLG* gene known as mammary gland cell growth factor (MGF) or stem cell factor (SCF). It encodes the ligand of c-Kit, a receptor tyrosine kinase, and participates in many biological processes, including hematopoiesis, gametogenesis, and melanogenesis (Talenti et al., 2018). The *KITLG* gene affects pigmentation in both humans and mice (Guenther et al., 2014). Polymorphisms in the *KITLG* gene have already been associated with litter size in goats (An et al., 2012, 2015). The genomic analysis suggested that *KITLG* is Responsible for a Roan Pattern in two Pakistani Goat Breeds (Talenti et al., 2018). Pigmentation genes *KITLG* have also been shown to have strong selection characteristics on Tibetan Cashmere Goat (Guo et al., 2019).

OARX_79209204.1 is within the *COL4A6* gene (Collagen Type IV Alpha 6 Chain), a protein-coding gene that encodes the alpha-6 chain of type IV collagen basal membranes. The genes *COL4A5* and *COL4A6* are located head-to-head near human chromosome Xq22. *COL4A6* activates transcription with 2 selectable promoters in a particular way of the tissue (Sugimoto et al., 1994). Gene Ontology (GO) annotations related to this gene include structural molecule activity and extracellular matrix structural constituent. Wang et al. (2018) suggested that the two dislocation mutations in *COL4A5* and *COL4A6* could be risk factors for cerebrovascular fibromuscular dysplasia. The dislocation mutation of the *COL4A6* gene causes serious non-syndromic hearing impairment in males (Rost et al., 2014). Downregulation of *COL4A6* may promote prostate cancer progression and invasion (Ma et al., 2020).

The 9 SNPs found in GWAS were verified by using 202 G3 generation ewes of the nucleus herd. Nine products were amplified around the 9 sites, and 29 SNPs were found using direct sequencing. Ovine SNP50 BeadChip developed by Illumina contains 54,241 SNP sites, with a mark on average every 46 kb. However, it is estimated that 1 SNP will appear every 1,000 bp in the human genome. Due to the insufficient SNP density of GWAS chips for commercial use, only 15% of genetic variation could be tested; thus, a large amount of genetic variation is yet to be found. Because of such GWAS defects, follow-up herd verification is essential. Herd verification is not only a supplement for the results of GWAS analysis but may also reveal new sites during the verification.

Our results revealed four new SNPs in follow-up verification that were significantly correlated to Hu sheep’s body size traits. The results of dual-luciferase reporter gene experiments showed that the 4 SNPs could significantly impact gene transcription activity. These significant sites can be included in our analysis field because they are close to GWAS positive sites. Therefore, we believe that GWAS is an important tool for candidate functional genes and the screening of functional SNP that can be used as a signpost to guide follow-up verification, thus preventing researchers from being overwhelmed by sequential information. However, a higher-density SNP detection chip may greatly improve the reliability of the results.

## Data Availability Statement

Data supporting this study has been deposited in GEO—accession number GSE152717.

## Ethics Statement

The animal study was reviewed and approved by the Ethical Committee of Zhejiang Academy of Agricultural Sciences.

## Author Contributions

YJ was in charge of the whole trial. XS designed the experiments. JJ completed the majority of the experiments and was a major contributor in writing the manuscript. HS and YC participated in sampling and laboratory analyses. JW for animal feeding and care. All authors read and approved the final manuscript.

## Conflict of Interest

The authors declare that the research was conducted in the absence of any commercial or financial relationships that could be construed as a potential conflict of interest.
