# Supplementary material for: The GWAS Analysis of Body Size and Population Verification of Related SNPs in Hu Sheep
Source: Front Genet. 2021 May 20;12:642552. doi: 10.3389/fgene.2021.642552 (PMC8173124; doi:10.3389/fgene.2021.642552)
Supplement: Supplementary file 1 [file Data_Sheet_1.doc]

Supplementary Materials

# Supplementary Tables

**Table S1.** Primers for PCR and direct sequencing

| SNPs | Nearest gene  Distance# (bp) | Chr | Primer sequence (5' → 3') | Annealing degree (℃) | Amplification product length (bp) |
| --- | --- | --- | --- | --- | --- |
| s55433.1 | *LOC101119639* +63923 | 5 | CCTTCTCCACCTCAGCTCA | 55 | 322 |
|  |  |  | CTGACCAGCTGGAAGAACTG |  |  |
| OAR5_99879334.1 | *MCTP1*  within | 5 | CTAGGAAGAAGTCGGAGTCACC | 55 | 404 |
|  |  |  | CGCTAATACACTGCCATTCCT |  |  |
| OARX_79209204.1 | *COL4A6*  within | 27 | GTTACTCACTCAGCCATGTCCA | 55 | 348 |
|  |  |  | GCATGACAACCCTAATTCCTACC |  |  |
| OAR23_3237800.1 | *ZNF516*  -368856 | 23 | CTTGGGTGTAGCGAGTTCTCT | 55 | 279 |
|  |  |  | GCCAGTGGTTGAAGAAGCC |  |  |
| s26859.1 | *SELENOF*  +22053 | 1 | TGACAGTGACCAAGGATCCC | 55 | 230 |
|  |  |  | CTGGTTGCCTCTGGGTTAAC |  |  |
| OAR3_132833292.1 | *KITLG*  within | 3 | CTATAGATGAGAGGTCAAGTGTTC | 55 | 477 |
|  |  |  | TGACTGATCTTCTGGACAAGT |  |  |
| OAR6_95218086.1 | *NPFFR2*  +114271 | 6 | GTCCTACTTGTGGTCTCTGTTT | 53 | 385 |
|  |  |  | GTGTTGCTGGCCTAATATCTCA |  |  |
| OAR1_164254640.1 | *CADM2*  within | 1 | GCTGGGATGAAAGAGATTAACCA | 53 | 439 |
|  |  |  | CATCCTTGTCAACTTCCCTGGGTT |  |  |
| OARX_120998827.1 | *PRR32*  +10161 | 27 | GAGCTTTACAACTGAATATCACCTT | 53 | 500 |
|  |  |  | GGTTGGTTTGTTGTTAGGTATATG |  |  |

**Table S2**. Descriptive statistical analysis of the phenotypic values of body size of G1 and G2 hu sheep

| Index | Number | Minium | Maxium | Mean | S.E.mean | Std.deviation | Variance |
| --- | --- | --- | --- | --- | --- | --- | --- |
| Body height(cm) | 240 | 58.00 | 72.00 | 62.17 | 0.22 | 3.27 | 10.74 |
| body length | 240 | 60 | 85 | 67.09 | 0.44 | 6.36 | 40.55 |
| Chest circumference(cm) | 240 | 71.00 | 90.00 | 78.58 | 031 | 4.46 | 19.96 |
| tail length | 240 | 9.00 | 12.00 | 10.64 | 0.05 | 0.76 | 0.579 |
| tail width | 240 | 9.00 | 13.00 | 10.81 | 0.05 | 0.83 | 0.697 |

**Table S3**. Descriptive statistical analysis of the phenotypic values of body size of G3 hu sheep

| Index | Number | Minium | Maxium | Mean | S.E.mean | Std.deviation | Variance |
| --- | --- | --- | --- | --- | --- | --- | --- |
| Body height(cm) | 202 | 66.00 | 85.00 | 76.20 | 0.22 | 3.06 | 9.38 |
| Chest circumference(cm) | 202 | 87.00 | 129.00 | 103.52 | 0.50 | 7.08 | 50.10 |

**Table S4.** SNPs associated with body size traits and mutation types in Hu sheep

| Nearest gene  Distance# (bp) | SNP loci name | Mutagenesis | Mutation type | |  |
| --- | --- | --- | --- | --- | --- |
| *LOC101119639* +63923 | mutation at 83 bp downstream of s55433.1 | G>A | transition |  | |
|  | mutation at 134 bp downstream of s55433.1 | G>C | transversion |  | |
| *MCTP1*  within | mutation at 69 bp upstream of OAR5_99879334.1 | T>C | transition |  | |
|  | OAR5_99879334.1 | A>G | transition |  | |
| *COL4A6*  within | mutation at 103 bp upstream of OARX_79209204.1 | G>A | transition |  | |
|  | mutation at 74 bp upstream of OARX_79209204.1 | G>A | transition |  | |
|  | OARX_79209204.1 | G>A | transition |  | |
|  | mutation at 32 bp downstream of OARX_79209204.1 | T>C | transition |  | |
|  | mutation at 57 bp downstream of OARX_79209204.1 | T>C | transition |  | |
| *ZNF516*  -368856 | OAR23_3237800.1 | G>A | transition |  | |
|  | mutation at 4 bp downstream of OAR23_3237800.1 | A>G | transition |  | |
|  | mutation at 43 bp downstream of OAR23_3237800.1 | G>A | transition |  | |
| *SELENOF*  +22053 | mutation at 19 bp upstream of s26859.1 | T>G | transversion |  | |
|  | mutation at 50 bp downstream of s26859.1 | G>C | transversion |  | |
|  | mutation at 81 bp downstream of s26859.1 | A>G | transition |  | |
| *KITLG*  within | mutation at 234 bp upstream of OAR3_132833292.1 | C>A | transversion |  | |
|  | mutation at 124 bp upstream of OAR3_132833292.1 | A>G | transition |  | |
|  | mutation at 20 bp upstream of OAR3_132833292.1 | G>T | transversion |  | |
|  | mutation at 10 bp upstream of OAR3_132833292.1 | A>T | transversion |  | |
|  | mutation at 55 bp downstream of OAR3_132833292.1 | G>A | transition |  | |
|  | mutation at 91 bp downstream of OAR3_132833292.1 | A>G | transition |  | |
| *NPFFR2*  +114271 | mutation at 44 bp upstream of OAR6_95218086.1 | G>A | transition |  | |
|  | OAR6_95218086.1 | C>T | transition |  | |
| *PRR32*  *+*10161 | mutation at 126 bp upstream of OARX_120998827.1 | A>C | transversion |  | |
|  | OARX_120998827.1 | C>T | transition |  | |
| *CADM2*  within | OAR1_164254640.1 | G>A | transition |  | |
|  | mutation at 149 bp downstream of OAR1_164254640.1 | C>A | transversion |  | |
|  | mutation at 192 bp downstream of OAR1_164254640.1 | *T*>*C* | transition |  | |
|  | mutation at 235 bp downstream of OAR1_164254640.1 | T>C | transition |  | |

**Table S5.** Genetic parameters of SNPs associated with body size traits in Hu sheep

| Nearest gene  Distance# (bp) | SNP loci name | Ne | SIC | H |  |
| --- | --- | --- | --- | --- | --- |
| *LOC101119639* +63923 | mutation at 83 bp downstream of s55433.1 | 1.1425 | 0.1890 | 0.1247 |  |
|  | mutation at 134 bp downstream of s55433.1 | **1.5809** | **0.1500** | **0.3675** |  |
| *MCTP1*  within | mutation at 69 bp upstream of OAR5_99879334.1 | 1.0664 | 0.1422 | 0.0623 |  |
|  | OAR5_99879334.1 | 1.0000 | 0.0000 | 0.0000 |  |
| *COL4A6*  within | mutation at 103 bp upstream of OARX_79209204.1 | 1.6378 | 0.5781 | 0.3894 |  |
|  | mutation at 74 bp upstream of OARX_79209204.1 | 1.6440 | 0.5806 | 0.3917 |  |
|  | OARX_79209204.1 | **1.0050** | **0.0173** | **0.0049** |  |
|  | mutation at 32 bp downstream of OARX_79209204.1 | 1.0050 | 0.0173 | 0.0049 |  |
|  | mutation at 57 bp downstream of OARX_79209204.1 | 1.0050 | 0.0173 | 0.0049 |  |
| *ZNF516*  -368856 | OAR23_3237800.1 | 1.9510 | 0.6805 | 0.4875 |  |
|  | mutation at 4 bp downstream of OAR23_3237800.1 | **1.2953** | **0.3886** | **0.2280** |  |
|  | mutation at 43 bp downstream of OAR23_3237800.1 | 1.2830 | 0.3792 | 0.2206 |  |
| *SELENOF*  +22053 | mutation at 19 bp upstream of s26859.1 | 1.3199 | 0.4069 | 0.2424 |  |
|  | mutation at 50 bp downstream of s26859.1 | 1.6931 | 0.5996 | 0.4094 |  |
|  | mutation at 81 bp downstream of s26859.1 | 1.2770 | 0.3744 | 0.2169 |  |
| *KITLG*  within | mutation at 234 bp upstream of OAR3_132833292.1 | 1.1425 | 0.2454 | 0.1247 |  |
|  | mutation at 124 bp upstream of OAR3_132833292.1 | 1.1369 | 0.2388 | 0.1204 |  |
|  | mutation at 20 bp upstream of OAR3_132833292.1 | 1.1369 | 0.2388 | 0.1204 |  |
|  | mutation at 10 bp upstream of OAR3_132833292.1 | 1.0100 | 0.0312 | 0.0099 |  |
|  | mutation at 55 bp downstream of OAR3_132833292.1 | 1.1369 | 0.2388 | 0.1204 |  |
|  | mutation at 91 bp downstream of OAR3_132833292.1 | 1.1258 | 0.2253 | 0.1118 |  |
| *NPFFR2*  +114271 | mutation at 44 bp upstream of OAR6_95218086.1 | 1.0980 | 0.1890 | 0.0892 |  |
|  | OAR6_95218086.1 | **1.0713** | **0.1500** | **0.0666** |  |
| *PRR32*  +10161 | mutation at 126 bp upstream of OARX_120998827.1 | 1.1088 | 0.2036 | 0.0981 |  |
|  | OARX_120998827.1 | 1.0505 | 0.1156 | 0.0480 |  |
| *CADM2*  within | OAR1_164254640.1 | 1.1197 | 0.2176 | 0.1069 |  |
|  | mutation at 149 bp downstream of OAR1_164254640.1 | **1.0713** | **0.1500** | **0.0666** |  |
|  | mutation at 192 bp downstream of OAR1_164254640.1 | 1.2277 | 0.3326 | 0.1855 |  |
|  | mutation at 235 bp downstream of OAR1_164254640.1 | 1.2277 | 0.3326 | 0.1855 |  |

SIC **:Shannon information content；**H:**Site heterozygosity**

**Table S6.** Population genetic analysis of SNPs related to body size traits in Hu sheep

| Gene | Gene loci name | | | Genetype | Numbers | | | Genetype frequencies | | | Allele | | | | Allele frequencies | | | PIC | |
| --- | --- | --- | --- | --- | --- | --- | --- | --- | --- | --- | --- | --- | --- | --- | --- | --- | --- | --- | --- |
| *LOC101119639* +63923 | mutation at 83 bp downstream of s55433.1 G>A | GG | | | | 176 | | | 0.8713 | | G | | 0.9332 | | | **0.1169** | | |  |
|  |  | GA | | | | 25 | | | 0.1237 | |  | |  | | |  |  |  |  |
|  |  | AA | | | | 1 | | | 0.0050 | | A | | 0.0668 | | |  |  |  |  |
|  | mutation at 113 bp downstream of s55433.1 G>C | GG | | | | 153 | | | 0.7574 | | G | | 0.7574 | | | **0.2999** | | |  |
|  |  | CC | | | | 49 | | | 0.2426 | | C | | 0.2426 | | |  |  |  |  |
| *MCTP1*  within | mutation at 69 bp upstream of OAR5_99879334.1 T>C | TT | | | | 189 | | | 0.9356 | | T | | 0.9678 | | | 0.0604 | | |  |
|  |  | TC | | | | 13 | | | 0.0644 | | C | | 0.0322 | | |  |  |  |  |
|  | OAR5_99879334.1 A>G | GG | | | | 202 | | | 1.0000 | | A | |  | | | 0 | | |  |
|  |  |  | | | |  | | |  | | G | | 1.0000 | | |  |  |  |  |
| *COL4A6*  within | mutation at 103 bp upstream of OARX_79209204.1 G>A | GG | | | | 114 | | | 0.5644 | | A | | 0.2649 | | | **0.3136** | | |  |
|  |  | GA | | | | 69 | | | 0.3416 | |  | |  | | |  |  |  |  |
|  |  | AA | | | | 19 | | | 0.0940 | | G | | 0.7351 | | |  |  |  |  |
|  | mutation at 74 bp upstream of OARX_79209204.1 G>A | GG | | | | 113 | | | 0.5594 | | A | | 0.2673 | | | **0.3150** | | |  |
|  |  | GA | | | | 70 | | | 0.3465 | |  | |  | | |  |  |  |  |
|  |  | AA | | | | 19 | | | 0.0941 | | G | | 0.7327 | | |  |  |  |  |
|  | OARX_79209204.1 G>A | GG | | | | 201 | | | 0.9950 | | A | | 0.0025 | | | 0.0050 | | |  |
|  |  | GA | | | | 1 | | | 0.0050 | | G | | 0.9975 | | |  |  |  |  |
|  | mutation at 32 bp downstream of OARX_79209204.1 T>C | TT | | | | 201 | | | 0.9950 | | C | | 0.0025 | | | **0.0050** | | |  |
|  |  | TC | | | | 1 | | | 0.0050 | | T | | 0.9975 | | |  |  |  |  |
|  | mutation at 57 bp downstream of OARX_79209204.1 T>C | TT | | | | 201 | | | 0.9950 | | C | | 0.0025 | | | **0.0050** | | |  |
|  |  | TC | | | | 1 | | | 0.0050 | | T | | 0.9975 | | |  |  |  |  |
| *ZNF516*  -368856 | OAR23_3237800.1 G>A | GG | | | | 67 | | | 0.3317 | | A | | 0.4208 | | | **0.3686** | | |  |
|  |  | GA | | | | 100 | | | 0.4950 | |  | |  | | |  |  |  |  |
|  |  | AA | | | | 35 | | | 0.1733 | | G | | 0.5792 | | |  |  |  |  |
|  | mutation at 4 bp downstream of OAR23_3237800.1 A>G | AA | | | | 154 | | | 0.7624 | | A | | 0.8688 | | | **0.2020** | | |  |
|  |  | GA | | | | 43 | | | 0.2129 | |  | |  | | |  |  |  |  |
|  |  | GG | | | | 5 | | | 0.0247 | | G | | 0.1312 | | |  |  |  |  |
|  | mutation at 43 bp downstream of OAR23_3237800.1 G>A | GG | | | | 155 | | | 0.7673 | | A | | 0.1262 | | | **0.1962** | | |  |
|  |  | GA | | | | 43 | | | 0.2129 | |  | |  | | |  |  |  |  |
|  |  | AA | | | | 4 | | | 0.0198 | | G | | 0.8738 | | |  |  |  |  |
| *SELENOF*  +22053 | mutation at 19 bp upstream of s26859.1 T>G | TT | | | | 148 | | | 0.7327 | | T | | 0.8589 | | | 0.2130 | | |  |
|  |  | TG | | | | 51 | | | 0.2525 | |  | |  | | |  |  |  |  |
|  |  | GG | | | | 3 | | | 0.0148 | | G | | 0.1411 | | |  |  |  |  |
|  | mutation at 50 bp downstream of s26859.1 G>C | GG | | | | 19 | | | 0.0941 | | C | | 0.7129 | | | 0.3256 | | |  |
|  |  | GC | | | | 78 | | | 0.3861 | |  | |  | | |  |  |  |  |
|  |  | CC | | | | 105 | | | 0.5198 | | G | | 0.2871 | | |  |  |  |  |
|  | mutation at 81 bp downstream of s26859.1 A>G | AA | | | | 154 | | | 0.7624 | | A | | 0.8762 | | | **0.1934** | | |  |
|  |  | GA | | | | 46 | | | 0.2277 | |  | |  | | |  |  |  |  |
|  |  | GG | | | | 2 | | | 0.0099 | | G | | 0.1238 | | |  |  |  |  |
| *KITLG*  within | mutation at 234 bp upstream of OAR3_132833292.1 C>A | CC | | | | 177 | | | 0.8762 | | A | | 0.0668 | | | 0.1169 | | |  |
|  |  | CA | | | | 23 | | | 0.1139 | |  | |  | | |  |  |  |  |
|  |  | AA | | | | 2 | | | 0.0099 | | C | | 0.9332 | | |  |  |  |  |
|  | mutation at 124 bp upstream of OAR3_132833292.1 A>G | AA | | | | 177 | | | 0.8762 | | A | | 0.9356 | | | 0.1132 | | |  |
|  |  | GA | | | | 24 | | | 0.1188 | |  | |  | | |  |  |  |  |
|  |  | GG | | | | 1 | | | 0.0050 | | G | | 0.0644 | | |  |  |  |  |
|  | mutation at 20 bp upstream of OAR3_132833292.1 G>T | GG | | | | 177 | | | 0.8762 | | G | | 0.9356 | | | 0.1132 | | |  |
|  |  | TG | | | | 24 | | | 0.1188 | |  | |  | | |  |  |  |  |
|  |  | TT | | | | 1 | | | 0.0050 | | T | | 0.0644 | | |  |  |  |  |
|  | mutation at 10 bp upstream of OAR3_132833292.1 A>T | AA | | | | 200 | | | 0.9901 | | A | | 0.9950 | | | 0.0099 | | |  |
|  |  | TA | | | | 2 | | | 0.0099 | | T | | 0.0050 | | |  |  |  |  |
|  | mutation at 55 bp downstream of OAR3_132833292.1 G>A | GG | | | | 177 | | | 0.8762 | | A | | 0.0644 | | | 0.1132 | | |  |
|  |  | GA | | | | 24 | | | 0.1188 | |  | |  | | |  |  |  |  |
|  |  | AA | | | | 1 | | | 0.0050 | | G | | 0.9356 | | |  |  |  |  |
|  | mutation at 91 bp downstream of OAR3_132833292.1 A>G | AA | | | | 178 | | | 0.8812 | | A | | 0.9406 | | | 0.1055 | | |  |
|  |  | GA | | | | 24 | | | 0.1188 | | G | | 0.0594 | | |  |  |  |  |
| *NPFFR2*  +114271 | mutation at 44 bp upstream of OAR6_95218086.1 G>A | | GG | | | | 1 | | | 0.0049 | | G | | 0.0468 | | | **0.0852** | | |
|  |  |  | GA | | | | 17 | | | 0.0837 | |  | |  | | |  |  |  |
|  |  |  | AA | | | | 185 | | | 0.9113 | | A | | 0.9532 | | |  |  |  |
|  | OAR6_95218086.1 C>T | | CC | | | | 192 | | | 0.9458 | | C | | 0.9680 | | | **0.0600** | | |
|  |  |  | CT | | | | 9 | | | 0.0443 | |  | |  | | |  |  |  |
|  |  |  | TT | | | | 2 | | | 0.0099 | | T | | 0.0320 | | |  |  |  |
| *PRR32*  +10161 | mutation at 126 bp upstream of OARX_120998827.1 A>C | | AA | | | | 182 | | | 0.8966 | | A | | 0.9483 | | | **0.0932** | | |
|  |  |  | AC | | | | 21 | | | 0.1034 | | C | | 0.0517 | | |  |  |  |
|  | OARX_120998827.1 C>T | | CC | | | | 193 | | | 0.9507 | | C | | 0.9754 | | | **0.0468** | | |
|  |  |  | CT | | | | 10 | | | 0.0493 | | T | | 0.0246 | | |  |  |  |
| *CADM2*  within | OAR1_164254640.1 G>A | | GG | | | | 181 | | | 0.8916 | | G | | 0.9433 | | | **0.1012** | | |
|  |  |  | GA | | | | 21 | | | 0.1034 | |  | |  | | |  |  |  |
|  |  |  | AA | | | | 1 | | | 0.0049 | | A | | 0.0567 | | |  |  |  |
|  | mutation at 149 bp downstream of OAR1_164254640.1 C>A  OAR1_164254640.1下游192bp T→C | | CC | | | | 189 | | | 0.9310 | | C | | 0.9655 | | | **0.0644** | | |
|  |  |  | CA | | | | 14 | | | 0.0690 | | A | | 0.0345 | | |  |  |  |
|  | mutation at 192 bp downstream of OAR1_164254640.1 T>C | | TT | | | | 163 | | | 0.8030 | | T | | 0.8966 | | | **0.1682** | | |
|  |  |  | TC | | | | 38 | | | 0.1872 | |  | |  | | |  |  |  |
|  |  |  | CC | | | | 2 | | | 0.0099 | | C | | 0.1034 | | |  |  |  |
|  | mutation at 235 bp downstream of OAR1_164254640.1 T>C | | TT | | | | 163 | | | 0.8030 | | T | | 0.8966 | | | **0.1682** | | |
|  |  |  | TC | | | | 38 | | | 0.1872 | |  | |  | | |  |  |  |
|  |  |  | CC | | | | 2 | | | 0.0099 | | C | | 0.1034 | | |  |  |  |

# Supplementary Figure

**Figure S1 Alignment of different genotypes amplification products of** **s554331 cloned into the pGL4.10 vector**

Genotype CC 1 CCTTCTCCACCTCAGCTCAGTCAGCGAGGGCCAAGTTTGTGGACTGCAGTCGCTGTCTCC
Genotype GG 1 CCTTCTCCACCTCAGCTCAGTCAGCGAGGGCCAAGTTTGTGGACTGCAGTCGCTGTCTCC
consensus 1 ************************************************************

Genotype CC 61 TGGCAGATGTTCTGCTCCGCTGTGTCACACTCAGCTAGGAAGGCCTGAATCCTTTTCACC
Genotype GG 61 TGGCAGATGTTCTGCTCCGCTGTGTCACACTCAGCTAGGAAGGCCTGAATCCTTTTCACC
consensus 61 ************************************************************

Genotype CC 121 AAACCCCTCCTTTTCATTCTACCGTCTCTGAAATTTTCTGGCAGAATTAGTGTGTCAATC
Genotype GG 121 AAACCCCTCCTTTTCATTCTACCGTCTCTGAAATTTTCTGGCAGAATTAGTGTGTCAATC
consensus 121 ************************************************************

Genotype CC 181 TCCTCCAAGATCTTCATAAACTGCTCGATCGTGGCTTTTACTCTCCTATCAAGTTTGCAG
Genotype GG 181 TCCTCCAAGATCTTCATAAACTGCTCGATCGTGGCTTTTACTCTCCTATCAAGTTTGCAG
consensus 181 ************************************************************

Genotype CC 241 AGAGCTTCAGTTTGCAAACCCTCGGCCAGAAAACCCTGCTGGATTCCAGCAAGGTCTTTA
Genotype GG 241 AGAGCTTCAGTTTGCAAACCGTCGGCCAGAAAACCCTGCTGGATTCCAGCAAGGTCTTTA
consensus 241 ******************** ***************************************

Genotype CC 301 TTCAGTTCTTCCAGCTGGTCAG
Genotype GG 301 TTCAGTTCTTCCAGCTGGTCAG
consensus 301 **********************

**Figure S2 Multiple alignment of different haplotypes amplification products of s26859.1 cloned into the pGL4.10 vector**

Haplotype_TTAA 1 TGACAGTGACCAAGGATCCCAGTTTGCCGGCCATGAAGGCCATTCCCATCGGGGATGGGC
Haplotype_GGAA 1 TGACAGTGACCAAGGATCCCAGTTTGCCGGCCATGAAGGCCATTCCCATCGGGGATGGGC
Haplotype_TTGG 1 TGACAGTGACCAAGGATCCCAGTTTGCCGGCCATGAAGGCCATTCCCATCGGGGATGGGC
Haplotype_GGGG 1 TGACAGTGACCAAGGATCCCAGTTTGCCGGCCATGAAGGCCATTCCCATCGGGGATGGGC
consensus 1 ************************************************************

Haplotype_TTAA 61 TGATAAAAAGTCATATACTAGTGTTTCCACCTGCCTCACCACCCTGCTGCTCCCAGACTA
Haplotype_GGAA 61 TGATAAAAAGTCATATACGAGTGTTTCCACCTGCCTCACCACCCTGCTGCTCCCAGACTA
Haplotype_TTGG 61 TGATAAAAAGTCATATACTAGTGTTTCCACCTGCCTCACCACCCTGCTGCTCCCAGACTA
Haplotype_GGGG 61 TGATAAAAAGTCATATACGAGTGTTTCCACCTGCCTCACCACCCTGCTGCTCCCAGACTA
consensus 61 ****************** *****************************************

Haplotype_TTAA 121 ATGGGAAGGATGAAGGGGCTACTTCAAGAACAGTGAGGCCCCCAACACATGGACACACAC
Haplotype_GGAA 121 ATGGGAAGGATGAAGGGGCTACTTCAAGAACAGTGAGGCCCCCAACACATGGACACACAC
Haplotype_TTGG 121 ATGGGAAGGATGAAGGGGCTACTTCAAGAACAGTGAGGCCCCCAACACATGGACACACGC
Haplotype_GGGG 121 ATGGGAAGGATGAAGGGGCTACTTCAAGAACAGTGAGGCCCCCAACACATGGACACACGC
consensus 121 **********************************************************.*

Haplotype_TTAA 181 CAGGAGCAATTGTCCCAGTGCCTGCGCAGTGTTAACCCAGAGGCAACCAG
Haplotype_GGAA 181 CAGGAGCAATTGTCCCAGTGCCTGCGCAGTGTTAACCCAGAGGCAACCAG
Haplotype_TTGG 181 CAGGAGCAATTGTCCCAGTGCCTGCGCAGTGTTAACCCAGAGGCAACCAG
Haplotype_GGGG 181 CAGGAGCAATTGTCCCAGTGCCTGCGCAGTGTTAACCCAGAGGCAACCAG
consensus 181 **************************************************
